# Construction of targeted ^10^B delivery agents and their uptake in gastric and pancreatic cancer cells

**DOI:** 10.3389/fonc.2023.1105472

**Published:** 2023-02-09

**Authors:** Song Wang, Zhengchao Zhang, Lele Miao, Jiaxing Zhang, Futian Tang, Muzhou Teng, Yumin Li

**Affiliations:** ^1^ Department of General Surgery, Second Hospital of Lanzhou University, Lanzhou, China; ^2^ Key Laboratory of the Digestive System Tumors of Gansu Province, Second Hospital of Lanzhou University, Lanzhou, China

**Keywords:** boron neutron capture therapy (BNCT), boron delivery agent, tumor, selectivity, solubility

## Abstract

Boron Neutron Capture Therapy (BNCT) is a new binary radiation therapy for tumor tissue, which kills tumor cells with neutron capture reaction. Boron neutron capture therapy has become a technical means for glioma, melanoma, and other diseases has been included in the clinical backup program. However, BNCT is faced with the key problem of developing and innovating more efficient boron delivery agents to solve the targeting and selectivity. We constructed a tyrosine kinase inhibitor-*L*-p-boronophenylalanine (TKI-BPA) molecule, aiming to improve the selectivity of boron delivery agents by conjugating targeted drugs while increasing the molecular solubility by adding hydrophilic groups. It shows excellent selectivity in differential uptake of cells, and its solubility is more than 6 times higher than BPA, leading to the saving of boron delivery agents. This modification method is effective for improving the efficiency of the boron delivery agent and is expected to become a potential alternative with high clinical application value.

## 1 Introduction

In the BNCT process, tumor cells are selectively allowed to accumulate boron drugs in advance, and then the tumor tissue is irradiated with neutron beam, causing ^10^B atoms to become unstable ^11^B atoms and then occur α-decay. Neutrons with great kinetic energy will attack ^10^B with insufficient neutrons and form ^11^B after capturing neutrons, while excessive energy cannot be carried by ^11^B, and then decay occurs (1–3). This process produces high-energy α-particle and ^7^Li recoil particle simultaneously release a large amount of energy in a short distance to selectively kill tumor cells (4–7). BNCT has conducted clinical studies on various diseases, including glioblastoma multiforme, primary and recurrent head and neck cancer, lung cancer, and hepatocarcinoma (8, 9). The boron delivery agent’s role is to transport ^10^B to the lesion and keep it stable. Boron delivery agent is generally required to meet the high selectivity requirements for tumor cells and can be efficiently ingested by them. At the same time, it is a basic requirement that the boron delivery agent is ingested less by normal cells (10, 11). Some researchers have proposed that the distribution ratio of boron delivery agents in tumor/blood or tumor/normal tissues should be greater than 3, and the boron delivery agent used can be considered as selective. As for the absolute amount of tumor intake, it is generally believed that ~20 μg ^10^B per gram of tissue or each tumor cell contains more than 10^9^ (1.66 × 10^-3^ pmol) ^10^B atoms reaches the lower limit of the amount of killer cells that can be killed during decay (12, 13). In addition, we hope to achieve relatively high enrichment in tumor cells and consume a small amount of boron delivery agent, meaning that the selectivity and solubility of boron delivery agent must be qualified (14). However, the boron delivery agents currently used are difficult to meet these requirements. They have more or less the disadvantages of poor selectivity and solubility (15), resulting in patients needing to be injected with several liters of drug solution within a few hours, which is a great burden for these endangered patients (16). This strategy needs to be adjusted.

BPA-fructose (BPA-F) complex is a hydrophilic chemical modification product of *L*-p-Boronophenylalanine (BPA), which is widely used in clinical. The introduction of water-soluble fructose into the structure can alleviate the poor solubility of BPA to some extent, but unfortunately, due to the difficulty of targeted delivery, a large number of drug solutions still need to be injected into the patients. Moreover, the preparation process of BPA-F will directly produce high concentration of sodium chloride. Considering the dosage of BPA-F, this scheme may be accompanied by the high osmotic pressure of the patient’s body, which is also one of the important factors that cannot be ignored.

Lapatinib is an oral small-molecule epidermal growth factor tyrosine kinase inhibitor, generally used for treating human epidermal growth factor receptor 2 (HER2)-positive breast cancer and gastric cancer (17, 18). Lapatinib inhibits the growth of tumor cells by binding to ATP binding sites in the receptor’s intracellular domain and blocking the activities of HER1 and HER2 tyrosine kinase (19). Its effect is relatively mild, and it is generally used with other drugs to enhance the therapeutic effect, but its targeting and inhibition of tumor cells is undoubtedly the core of the procedure. In this study, we covalently coupled BPA with lapatinib to form a stable structure Lap-BPA and used the targeting ability of lapatinib to deliver drugs to the various tumor cells. At the same time, several hydrophilic groups of different degrees were introduced in the preparation process, which also improved the molecular solubility to a certain extent. We determined that the uptake of Lap-BPA by tumor cells and normal cells was significantly different, and the dosage was less, with no toxic or side effects observed within 24 hours. Lap-BPA shows satisfactory potential and is expected to become a clinical candidate molecule. In this work, we focused on improving the targeting of BPA, so that it can be better absorbed by tumor cells and achieve targeted delivery through drug loading, this is our main purpose.

## 2 Materials and methods

### 2.1 Source of materials

^10^B-labeled BPA (^10^B-BPA, abbreviated as BPA in the text) was provided by Professor Weiqiang Chen (Institute of Modern Physics and Key Laboratory of Heavy Ion Radiation Biology and Medicine, Chinese Academy of Sciences, Lanzhou, China). Other synthetic materials and solvents are purchased from Innochem, Beijing, China. Cell counting kit-8 (CCK-8) was obtained from Dojindo Molecular Technologies, RPMI 1640 Medium, DMEN Medium, trypsin-EDTA (0.05%) and Fetal bovine serum (FBS) were purchased from Gibco™. In the liquid chromatography experiment, column: Chiralpak AD-RH 150 * 4.6mm I.D., 5um.

### 2.2 Synthesis of Lap-BPA

#### 2.2.1 Synthesis of compound 2

To a solution of compound 1 (100 mg, 0.480 mmol) in EtOH (10 mL) and H_2_O (2 mL) was added K_2_CO_3_ (199 mg, 1.44 mmol) and Boc_2_O (157 mg, 0.720 mmol). The reaction was stirred at 25°C for 15 h. LC-MS showed the reaction was completed. N, N-dimethylethylenediamine (50 mg) was added to the reaction mixture and then stirred at 25°C for 1 h. The reaction mixture was diluted with water (20 mL) and acidified by 1N HCl to pH=1. The mixture was extracted with EtOAc (20 mL x2). The combined organic layer was washed with brine (25 mL), dried over sodium sulfate and concentrated under reduced pressure to give compound 2 (148 mg, yield: 100%) as a white solid. LC-MS: t_R_ = 0.544 min, MS (ESI) m/z = 253.1 [M+H-t-Bu]^+^. ^1^H NMR (400 MHz, DMSO-d_6_) δ = 12.59 (br s, 1H), 7.96 (s, 2H), 7.69 (d, J=8.0 Hz, 2H), 7.20 (d, J=8.0 Hz, 2H), 7.09 (d, J=8.4 Hz, 1H), 4.15 - 4.05 (m, 1H), 3.01 (dd, J=4.4, 13.6 Hz, 1H), 2.82 (br dd, J = 10.4, 13.6 Hz, 1H), 1.33 (s, 9H).

#### 2.2.2 Synthesis of compound 4

To a solution of compound 3 (200 mg, 0.344 mmol) and compound 2 (127 mg, 0.413 mmol) in DMF (10 mL) was added DIPEA (148 mg, 1.15 mmol) and HATU (196 mg, 0.516 mmol). The reaction mixture was stirred at 25°C for 15 h. LC-MS showed the reaction completed. The reaction mixture was poured into water (50 mL) and extracted with EtOAc (25 mL x 2). The combined organic extracts were washed with brine (20 mL x2), dried over sodium sulfate and concentrated under reduced pressure to give compound 3 (300 mg, crude) as yellow gum, which was used directly in the next step without further purification. LC-MS: t_R_ = 0.723 min, MS (ESI) m/z = 871.3 [M+H]^+^.

#### 2.2.3 Synthesis of compound 5

To a mixture of compound 4 (300 mg, crude) in dioxane (10 mL) was added HCl/dioxane (2 mL, 4 M), the reaction mixture was stirred at 25°C for 2 h to give a yellow solution. LC-MS showed the reaction was completed. The mixture was concentrated under reduced pressure to give a residue. Prep-HPLC purified the residue (column: Boston Prime C18 150*30mm*5um; mobile phase: [water (NH_3_H_2_O+NH_4_HCO_3_)-ACN]; B%: 43%-73%, 10min) to give T.M.5 (150 mg, yield: 56%, ee: 97.64%) as a yellow solid. LC-MS: t_R_ = 1.896 min, mobile phase: A: water with 0.069% TFA, B: acetonitrile, gradient: from 10% to 80% of B in A in 11 min, then from 80% to 10% in 1 min, and hold 10% for 8 min. MS (ESI) m/z = 771.1 [M+H]^+^. ^1^H NMR (400 MHz, DMSO-d_6_) δ = 9.85 (br d, J=16.7 Hz, 1H), 8.73 (s, 1H), 8.56 (s, 1H), 8.15 - 8.07 (m, 1H), 8.01 (d, J=2.4 Hz, 1H), 7.96 - 7.91 (m, 2H), 7.83 - 7.77 (m, 1H), 7.75 - 7.64 (m, 3H), 7.53 - 7.44 (m, 1H), 7.36 - 7.27 (m, 3H), 7.23 - 7.13 (m, 3H), 7.08 - 7.01 (m, 1H), 6.51 - 6.32 (m, 1H), 5.26 (s, 2H), 4.77 - 4.66 (m, 1H), 4.63 - 4.53 (m, 1H), 4.12 - 3.86 (m, 1H), 3.79 - 3.63 (m, 2H), 3.51 (td, J=7.1, 14.1 Hz, 1H), 3.28 - 3.22 (m, 1H), 3.04 - 2.97 (m, 3H), 2.96 - 2.89 (m, 1H), 2.78 - 2.64 (m, 1H), 1.95 (br s, 2H). ^13^C NMR (100 MHz, DMSO-d_6_) δ = 44.605, 51.560, 52.372, 52.747, 52.870, 69.866, 108.286, 111.304, 114.406, 114.621, 114.789, 115.073, 115.280, 115.762, 121.545, 122.893, 123.797, 124.716, 128.339, 128.998, 130.997, 131.073, 134.505, 140.073, 140.150, 149.433, 150.268, 151.647, 151.976, 152.926, 154.872, 158.020, 161.466, 163.887, 175.039, 175.231.

### 2.3 Cytotoxicity of Lap-BPA against tumor and normal cell

Various cells were inoculated into 96-well culture plates at the density of 1×10^4^ cells per well, and the cells were incubated with Lap-BPA (containing 0.01 ‰ DMSO) of various concentrations for 24 and 48 hours at 37 °C and 5% CO_2_, respectively. After incubation, the cell culture medium was removed and the cells were exposed to CCK-8 (100 µl, V CCK 8: V DMEM= 1:9). Then, the absorbance was measured at 450nm (Elx800, BioTek) using a microplate reader. Cell viability is calculated as (At-Anc)/(Apc-Anc) × 100%, where At, Apc, and Anc represent the absorbance of the test, positive control, and negative control groups, respectively.

### 2.4 Determination of ^10^B level

This experiment referred to the previous literature (20). After removing the culture medium (1 × 10^5^ various cells, 1 ml culture medium), wash the cells with PBS three times, then add 500 μl trypsin to digest the cells, then take out all the cells, add 2 ml aqua regia (HCl/HNO_3_, v/v = 3:1, 2.0 mL), and heat to 80 °C for 3 hours until a clear solution is obtained. Then dilute the solution to 3 ml with deionized water for ICP-AES analysis. Then we dilute the sample. In this process, we will use deionized water. After absorbing the mother liquor, we will mix the sample with deionized water, filter it, take 1ul from it, place it on the surface of the sample box, and send it to the instrument to select boron for testing.

### 2.5 Correlation curve between integral area and BPA and Lap-BPA concentration

Dissolve Lap-BPA in DMSO to make 100 mM storage solution, and dilute it with PBS to different concentrations. Dissolve BPA in 1 M NaOH (aq.), adjust the pH to about 7 with 1 M HCl (aq.), add deionized water to prepare storage solution, and dilute it to different concentrations. The relationship between the corresponding integral area and the concentration was obtained by liquid chromatography and repeated three times.

### 2.6 Western blot analysis

The cells were dissolved in 1% Triton lysis buffer and quantified by Kaumas blue method. Cell lysates were isolated by SDS-PAGE and transferred to PVDF membrane. PVDF membrane was blocked with 5% skim milk powder in TBST buffer solution at room temperature for 2 hours. Then incubate the membrane with primary antibodies (HER2 1:1000, Actin 1:1000) at 4 °C overnight, and then incubate it with secondary antibodies (CST, 1:2000) at room temperature for 60 minutes. Protein was detected with SuperSignal Western Pico Chemiluminescence Substrate; Pierce, USA, and visualized with electrophoresis gel imaging analysis system (Hercules, California, USA).

### 2.7 HER2 siRNA

Three siRNAs targeting HER2 and one scrambled control were purchased from VIEWSOLID BIOTECH (Beijing, China). Cells with 5 × 10^5^ cells/well in 6-well plate. Transfect cells with siRNA according to the manufacturer’s instructions.

### 2.8 Statistical analysis

The data are expressed as mean ± SD of at least three independent experiments. Student’s t-test using GraphPad Prism software version 3.06 (La Jolla, California, USA) was used for statistical analysis of data; When p<0.05, the difference between groups was considered statistically significant.

## 3 Results and discussion

### 3.1 The structure of Lap-BPA

**Figure 1:**
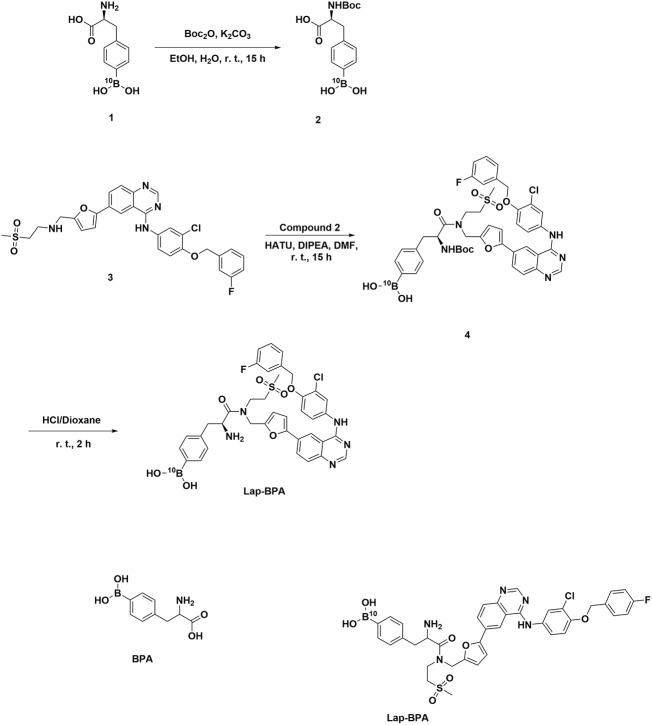
Synthesis of Lap-BPA.

Previous studies on BNCT have often focused on superficial tumors. For deep tumors, it is difficult to intervene through BNCT due to the difficulties in drug administration and radiation depth (14, 21). Within our capabilities, we can solve the problem of improving the properties of boron delivery agents. In this work, we guided BPA close to tumor cells with the selectivity of targeted drugs to tumors, and firmly combined the whole molecule containing BPA with tumor cells through targeted drugs. The advantages of this method are that the molecular volume after modification remains small, and BPA is difficult to eliminate the selectivity of targeted drugs; in terms of function, molecules are divided into different units, each of which has a function; after coupling, strong polar bonds will be formed, or the solubility of molecules can be improved, especially for BPA. This design method has never been used before, and it is also an attempt made by us in the new boron delivery agent, with the purpose of better improving its effect. We have tried a variety of approved drugs, including imatinib, sorafenib, lapatinib and other chemotherapy drugs, which have been included in our program, but unfortunately, only lapatinib has shown better solubility. Therefore, we only discussed lapatinib-modified BPA in this paper. We synthesized Lap-BPA according to the method in **Figure 1**. Compared with other boron delivery agents, such as targeted boron delivery agents CuTCPH (22) and [K_4_-Nε (Cpa)]-[F^7^, P^34^]-NPY (23) generally requires complex preparation process, including metal complexation or protein recombination. Lap-BPA has a simple preparation method, it does not use highly toxic substances in the preparation process and can be obtained quickly. For the molecular design, we chose amide as the bond connecting lapatinib and BPA, because it can keep relatively stable under physiological conditions and is not easy to be hydrolyzed by alkaline pH or other enzymes. In addition, the blocking of fatty amino group can reduce the flexibility of lapatinib end, which may reduce the toxicity of the whole molecule to a certain extent without interfering with the binding of aromatic ring and receptor protein. In addition, we only modify the heteroatom of lapatinib a little, which will reduce its binding efficiency with the receptor.

### 3.2 Cytotoxicity of Lap-BPA

**Figure 2:**
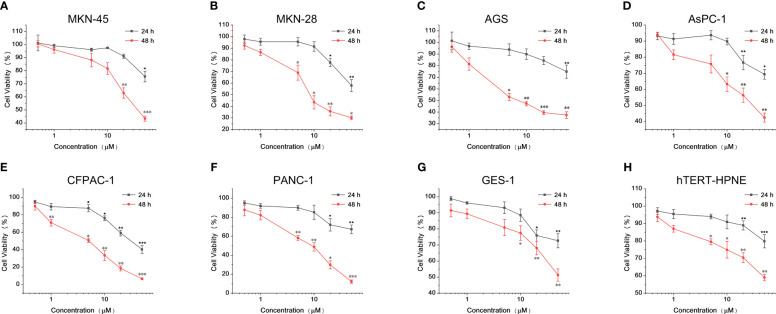
Cytotoxicity of Lap-BPA on co-culture of MKN-45, MKN-28, AGS, AsPC-1, PANC-1, CFPAC-1, GES-1 and hTERT-HPNE cells. Cytotoxicity of Lap-BPA to gastric cancer cells, including MKN45 **(A)**, MKN28 **(B)** and AGS cells **(C)**. Cytotoxicity of Lap-BPA to pancreatic cancer cells, including AsPC-1 **(D)**, CFPAC-1 **(E)** and PANC-1 cells **(F)**. The toxicity of Lap-BPA to normal cells, including GES-1 **(G)** and hTERT-HPNE cells **(H)**. *p < 0.05; **p < 0.01; ***p < 0.001; two-way ANOVA followed by Tukey’s multiple comparisons test.

We chose gastric cancer and pancreatic cancer, which are difficult to prevent and cure in the tumor (24, 25), as the research objects. Because they are not easy to remove in advanced cancer, they are more suitable for BNCT. More importantly, compared with the classic BNCT treatment objects (such as head and neck cancer and melanoma), it is not suitable to inject drugs in large doses for tumors in deep tissues, which will cause serious tissue damage. Therefore, it is more suitable to use low concentration of drugs. First, we need to determine the cytotoxicity of Lap-BPA, which includes not only different tumor cells, but also normal cells. We co-incubated Lap-BPA with several tumor cells, including gastric cancer cells MKN-45, MKN-28, AGS, pancreatic cancer cells AsPC-1, PANC-1, CFPAC-1, and human gastric epithelial cells GES-1 and human pancreatic duct-derived cells hTERT-HPNE. To observe the phenomenon of Lap-BPA incubating with different cells for 24 hours, Lap-BPA has almost no toxicity to several gastric cancer cells within 10 μM. When the concentration exceeds 20 μM, it has slight toxicity to three gastric cancer cells (**Figures 2A–C**). In pancreatic cancer cells, Lap-BPA co incubated with CFPAC-1 cells produced significant toxicity only at 1 μM, indicating that CFPAC-1 cells were not tolerant to Lap-BPA. It also did not produce obvious toxicity to Aspc-1 and PANC-1 cells when the concentration was within 20 μM (**Figures 2D–F**). According to the cell survival within 48 hours, most tumor cells have died to vary degrees, we listed the IC_50_ values of various tumor cells treated with Lap-BPA for 48 hours (**Table 1**). Almost from 5 μM, tumor cells can no longer withstand Lap-BPA killing. It may be because cells can still induce cells to produce living oxygen after combining with modified lapatinib, leading to cell death (26, 27), suggesting that our intervention time can be controlled within 24 hours. For GES-1 and hTERT-HPNE cells, the data within 24 hours showed that Lap-BPA had almost no cytotoxicity, only slight toxicity after 48 hours of high concentration of Lap-BPA (**Figures 2G, H**), which is undoubtedly an excellent reflection of the selectivity of Lap-BPA. Similarly, this result is more consistent with the evaluation principle of boron delivery agent, which is the guarantee of biological safety.

**Table 1:** IC_50_ value of drugs on various tumor cells within 48 hours.

cell	AGS	AsPC-1	CFPAC-1	MKN-28	MKN-45	PANC-1
IC_50_ (μM)	6.88 ± 0.67	12.29 ± 1.55	5.27 ± 0.28	8.74 ± 1.25	12.67 ± 2.31	9.55 ± 2.03

For the Lap-BPA co-incubated with GES-1 and hTERT-HPNE cells, we set the maximum concentration at 50 μM, which is consistent with other tumor cells, and there is no half inhibition within 48 hours.

Admittedly, BPA is widely used in clinical, but it is undeniable that its dosage is indeed large for each patient, which is determined by its own nature. From the perspective of structure, BPA is an analog of phenylalanine (Phe), which also has some functions of Phe, and there are reports about phenylalanine as a tumor drug carrier (28, 29). However, because BPA has a phenylboronic acid structure at the end of the molecule, it is easy to form intermolecular hydrogen bonds, that is, its own dimer, in the water phase or other proton solvents, which is very common in organic catalytic reactions (30), resulting in a decrease in its solubility compared with phenylalanine. Some studies have pointed out that the trend of hydrogen bonding and intermolecular complexation between amino acid end and boric acid end is obvious, and the product is relatively stable (31). The coordination between nitrogen atom and boron atom is due to the lack of electron property of boron atom, while boric acid will form ester with amino acid end. These processes will lose the proton, which will reduce the molecular polarity to a certain extent (32, 33). It is obvious that the elimination of active protons will affect its solubility. We list the structural characteristics of BPA and discuss the reasons for the decrease of its solubility in order to explain why it is used in large quantities in clinical. Due to the introduction of lapatinib fragment, the polymerization of BPA is hindered in space. A large number of heteroatoms and functional groups distributed in the lapatinib could repel adjacent BPA fragments in different forms, making Lap-BPA solvation easier.

### 3.3 The distribution of Lap-BPA in various cells

**Figure 3:**
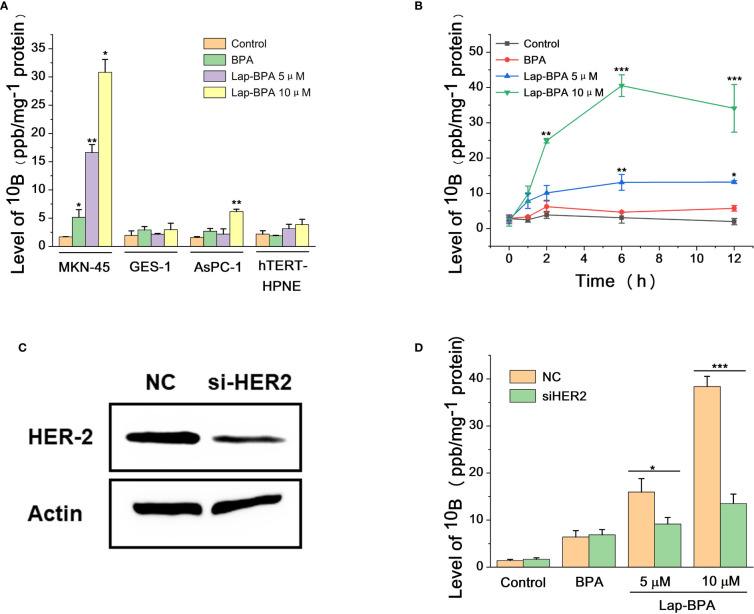
The uptake of ^10^B by tumor cells and normal cells. **(A)** After different concentrations of Lap-BPA and MKN-45, GES-1, AsPC-1 and hTERT-HPNE cells, the level of ^10^B in these cells was detected by ICP-AES, respectively, with 1 mg of total protein as the internal reference. Concentration of compounds co incubated with cells: BPA, 10 μM; Lap-BPA, 5 μM and 10 μM. **(B)** Different concentrations of Lap-BPA were co-incubated with MKN-45 cells, and the level of ^10^B in MKN-45 cells changed with the co-incubation time. **(C)** Western blot analysis of MKN-45 cells transfected with siRNA targeting HER2. **(D)** The ^10^B level of MKN45 cells transfected with siRNA targeting HER2 after co-incubation with various concentrations of Lap-BPA. Data are presented as mean ± SD determined by Student’ s t-test. *p < 0.05, **p < 0.01, *** p < 0.001 vs control group.

After determining the cytotoxicity of Lap-BPA, we need to test the efficacy of Lap-BPA, that is, the ^10^B content of each cell after incubating Lap-BPA with different cells. We selected MKN-45, GES-1, AsPC-1 and hTERT-HPNE cells, incubated them with drugs with concentrations of 5 μM and 10 μM, and then tested the drug content in different cells through inductively coupled plasma atomic emission spectroscopy (ICP-AES). Only MKN-45 cells at these two concentrations had significant differences compared with the control group, 7.2 and 15 times their levels, respectively, and 3.8 and 6 times of BPA treatment. Although the normal level of MKN-45 was also increased about twice after BPA (10 μM) treatment, the difference was insignificant or even lower among the other three cells (**Figure 3A**), suggesting that BPA may not be suitable for gastric and pancreatic cancer. It is a definite conclusion that MKN-45 cells are considered HER2 positive, and there is no doubt that lapatinib has high targeting for HER2 (34, 35). After treatment of MKN-45 cells with Lap-BPA, MKN-45 cells have high levels of ^10^B, which may be related to the targeting ability of lapatinib, so that Lap-BPA can be selectively absorbed by MKN-45 cells. By measuring the boron level of different BPA concentrations, we drew a correlation curve (**Supplementary Figure 8**), and obtained the absolute ^10^B concentration in four cells (10 μM Lap-BPA), which are 11.31 × 10^-3^ pmol (MKN-45), 1.83 × 10^-3^ pmol (AsPC-1), 1.01 × 10^-3^ pmol (hTERT-HPNE) and 0.79 × 10^-3^ pmol (GES-1) per cell. These are the concentrations of boron in different kinds of cells, for MKN-45 cells, this has exceeded the generally accepted standard that can be stimulated to decay, and the dosage is far less than BPA, this concentration of drugs is enough to cause decay under neutron irradiation. The absolute content of BPA (also known as ^10^B content, each BPA contains only one ^10^B atom) in these cells were 1.068 × 10^-3^ pmol (MKN-45), 0.36 × 10^-3^ pmol (AsPC-1), 0.42 × 10^-3^ pmol (hTERT-HPNE) and 0.28× 10^-3^ pmol (GES-1) per cell, respectively. Obviously, such ^10^B level is not enough to allow the fission of boron atoms to destroy cells. And it is also the most critical part for tumor cells to achieve targeted killing. To further verify the retention of Lap-BPA in MKN-45 cells, we selected MKN-45 cells treated with Lap-BPA in different lengths of time, and detected their ^10^B level after splitting them. For the cells treated with Lap-BPA, the ^10^B level of cells increased with time, and almost did not increase after 6 hours (**Figures 3B**), indicating that cells could complete the uptake of Lap-BPA within 6 hours. However, after treatment with high concentration of Lap-BPA, the ^10^B level of cells decreased slightly about 12 hours, which may be related to cell proliferation. In contrast, BPA treated cells, the element level only rose slightly within 12 hours, and there was no difference compared with control, which means that BPA could not be well absorbed by MKN-45 cells, which is consistent with our previous conclusion.

We set the concentration of Lap-BPA at the level of micromol per liter, which is a very small order of magnitude compared with previous works, thanks to the interaction between BPA and lapatinib after coupling. Lap-BPA has such an effect for various reasons. On the one hand, the lapatinib fragment gives better targeting, could bind to the receptor on the surface of MKN-45 cells, leading to the enrichment of BPA fragment to tumor cells. We still need to explain the way of Lap-BPA enrichment to tumor cells, which will be based on reducing the level of HER2. We constructed MKN-45 cells with HER2 knockdown (**Figures 3C**), treated them with different concentrations of Lap-BPA, and also compared the groups treated with BPA. The level of boron decreased significantly no matter the drug concentration was 5 μM or 10 μM, but the BPA treated group remained basically unchanged, indicating that the uptake of BPA by MKN-45 cells may be less affected by HER2 (**Figures 3D**). However, the level of HER2 has a great impact on the Lap-BPA treatment group, meaning that one of the key factors affecting Lap-BPA enrichment in tumor cells may be the level of HER2. In addition, introducing BPA fragments will change the spatial structure of lapatinib fragments, resulting in a certain degree of reduction of lapatinib receptor binding activity. These conclusions support that Lap-BPA is a ^10^B delivery agent that can target MKN-45 cells at a very low concentration.

### 3.4 Stability of Lap-BPA under physiological conditions

**Figure 4:**
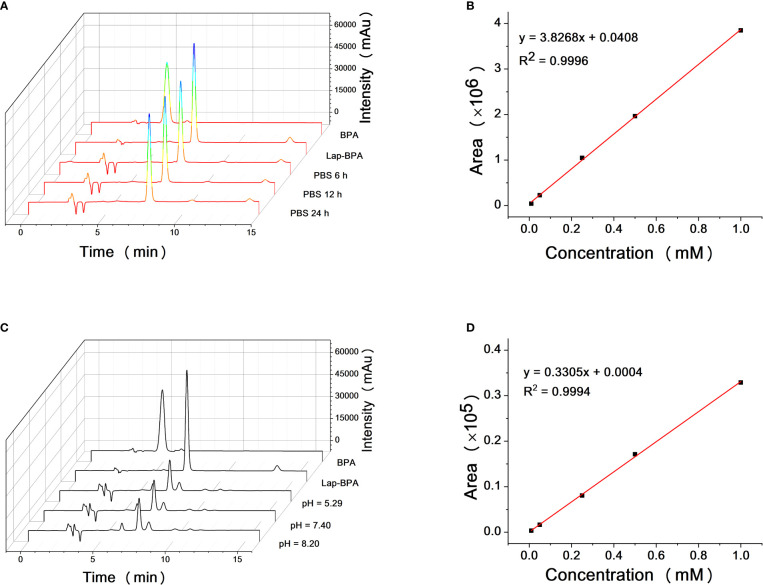
Solubility and structural stability of Lap-BPA under physiological conditions. **(A)** The retention time of Lap-BPA after dissolution in PBS. Correlation curve of integral area with Lap-BPA concentration **(B)** and BPA **(D)** concentration. **(C)** The structural changes of Lap-BPA after 24 hours of mixing in buffer solutions with various pH value.

It has been proved that Lap-BPA can target MKN-45 cells and transport boron, so we need to test the stability of Lap-BPA under physiological conditions. The excessive use of BPA has always been a difficult problem to solve. In application, the concentration of BPA is generally set at more than 100 mg/kg and needs to be completed within a few hours (36–39), meaning that patients need to be injected with several liters of liquid during this period, which is undoubtedly a great pressure. More importantly, the process of preparing BPA solution requires the use of high concentration hydrochloric acid and sodium hydroxide solution. Adjusting the pH will produce a large amount of sodium chloride (40–42). which is a challenge to the human body’s osmotic pressure, especially for the candidate patients with body failure, it is difficult to bear this pain, which is also one of the important obstacles to the development of BNCT. First, we tried to dissolve Lap-BPA in phosphate buffer saline (PBS) and mix it for 6, 12 and 24 hours respectively. Lap-BPA exists stably in PBS without obvious deterioration, and its structure remains stable within 12 hours as time goes on (**Figures 4A**), suggesting that Lap-BPA is a boron delivery agent with stable structure, and will not decompose or react with other substances under physiological pH. Next, we tested the stability of Lap-BPA in different pH buffers (**Figures 4C**). There was no apparent new peak in the liquid chromatography of Lap-BPA, indicating that Lap-BPA can remain stable within a small range of fluctuations near the physiological pH, and the amide bond that maintains the coupling is difficult to hydrolyze within 24 hours.

We have proved that Lap-BPA is a molecule with stable structure under physiological conditions, and continue to determine the precise concentration of Lap-BPA and BPA in the direct preparation process.

We obtained the concentration peak area curves of BPA and Lap-BPA using the classical preparation method (**Figures 4B–D**), then directly dissolved BPA and Lap-BPA in PBS without dilution, and then constant the volume to the accurate concentration, calculate the precise concentration of BPA and Lap-BPA through the correlation curve. The precise concentrations of BPA and Lap-BPA were calculated according to the correlation curve (**Table 2**). No matter how much BPA is added, BPA always maintains a very low concentration in PBS, indicating that BPA solution is difficult to be prepared by means of no sodium chloride formation. The exact concentration of Lap-BPA is close to the theoretical concentration, suggesting that Lap-BPA is relatively ideal in solubility.

**Table 2:** The exact concentrations of BPA and Lap-BPA in PBS were determined by liquid chromatography.

Concentration (μM)	1	5	10	20	50	100
BPA	–	0.79 ± 0.34	1.34 ± 0.26	1.37 ± 0.74	2.05 ± 1.19	3.51 ± 1.02
Lap-BPA	0.82 ± 0.13	4.10 ± 0.59	9.37 ± 0.35	17.44 ± 1.19	43.68 ± 4.61	82.47 ± 10.86

In the process of preparing solution, directly dissolve the substance in PBS to reach the target concentration without dissolving the substance in strong acid or alkali in advance. “-” indicates that the group is below the detection limit. The data were measured at 20 °C and repeated three times. Mobile phase: A: water with 0.1% TFA (65%) B: MeOH (35%), temp.: 20 °C.

## 4 Conclusion

We coupled BPA with lapatinib through amide bond to obtain boron delivery agent Lap-BPA, which showed good targeting ability in the transport process, and almost did not produce cytotoxicity within 24 hours. At the same time, the targeted transport of MKN-45 cells can be completed with a small dose, so the cells have a high level of ^10^B. Lap-BPA has good solubility and stability, is difficult to be hydrolyzed in general experimental procedures, and has a stable structure, so it has high clinical application potential. As for BPA containing ^10^B, it is so rare that each treatment will cost a lot on boron delivery agent. Due to the limitation of its properties, the part not absorbed by the human body dominates, resulting in much waste and burden on patients. Lap-BPA is a targeted variant of BPA, which endows BPA with new characteristics. This strategy will be adopted in molecular design to make BNCT more widely used.

## Data availability statement

The original contributions presented in the study are included in the article/**Supplementary Material**. Further inquiries can be directed to the corresponding authors.

## Author contributions

Conceptualization: MT and SW. Methodology: SW. Validation: SW, MT and ZZ. Formal analysis: MT and JZ. Investigation, SW. Resources: SW, LM and FT. Writing—original draft preparation: SW. Writing—review and editing: SW. Supervision: SW. Project administration: YL. All authors have read and agreed to the published version of the manuscript. All authors contributed to the article and approved the submitted version.
