# Supplementary material for: Construction of targeted 10B delivery agents and their uptake in gastric and pancreatic cancer cells
Source: Front Oncol. 2023 Feb 9;13:1105472. doi: 10.3389/fonc.2023.1105472 (PMC9947830; doi:10.3389/fonc.2023.1105472)
Supplement: Supplementary file 1 [file DataSheet_1.pdf]

Supplementary Material

Construction of targeted ^{10}B delivery agent and selective uptake by tumor cells

Identification of Lap BPA and its intermediates.

^{1}H-NMR, ^{13}C-NMR and ESI-MS Spectra

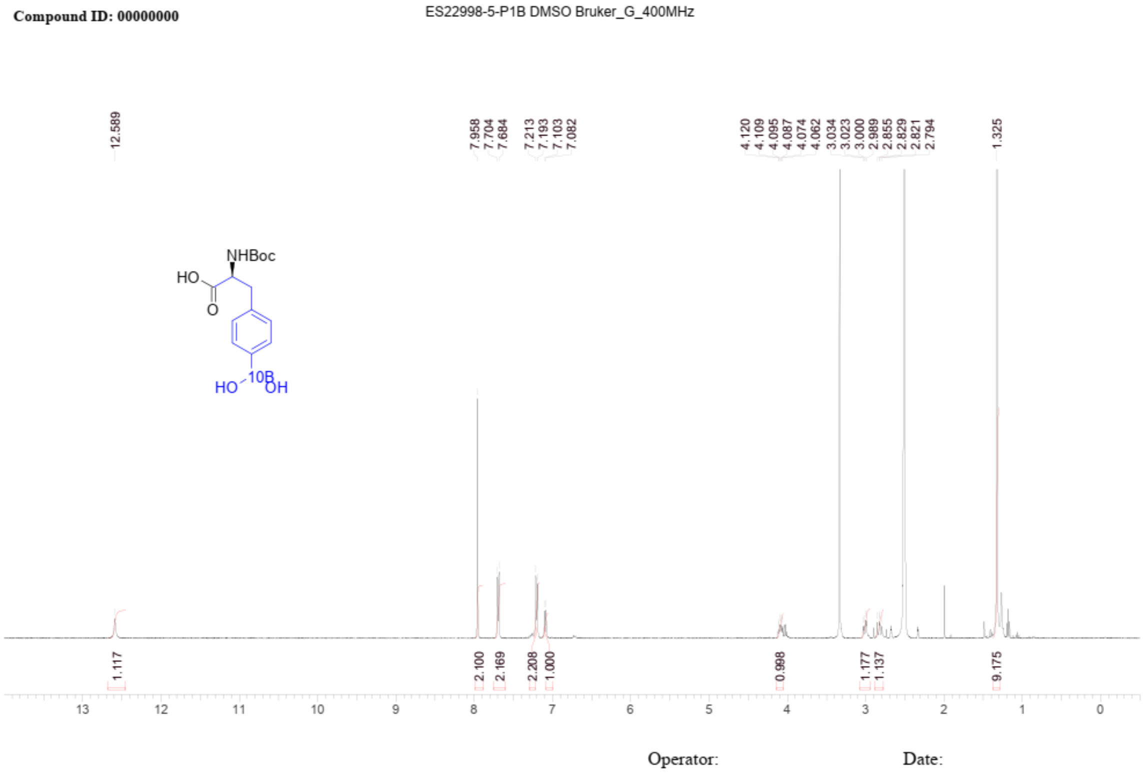

Supplementary Figure 1. ^{1}H-NMR spectrum of compound 2 in DMSO-d_6.

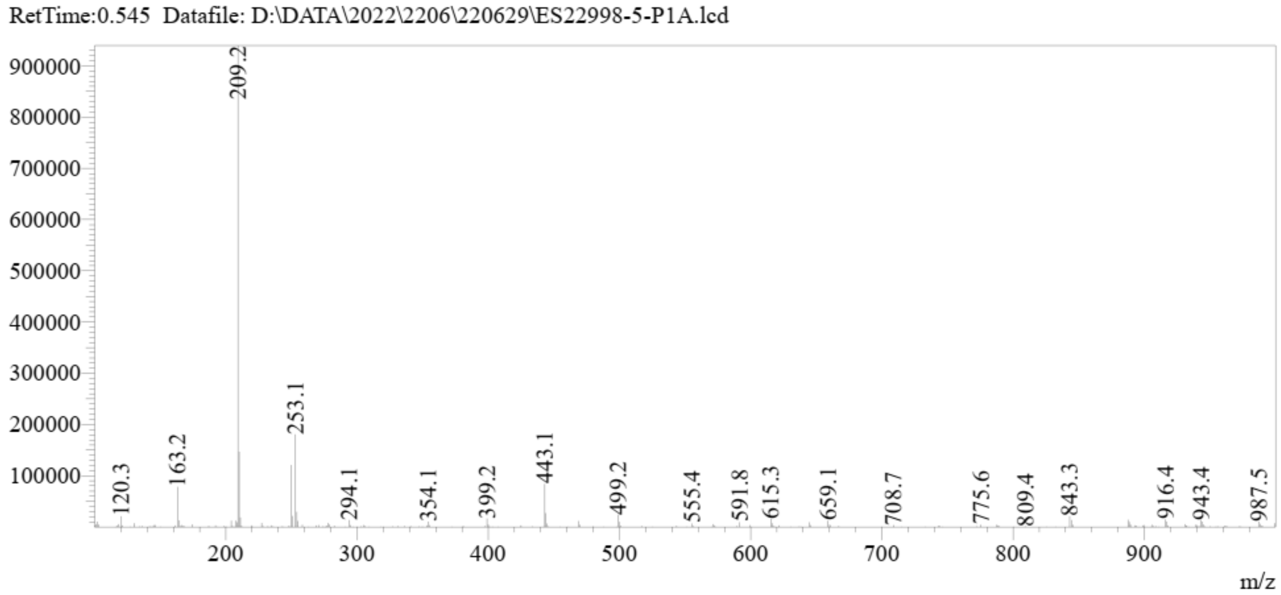

Supplementary Figure 2. ESI-MS spectrum of compound 2.

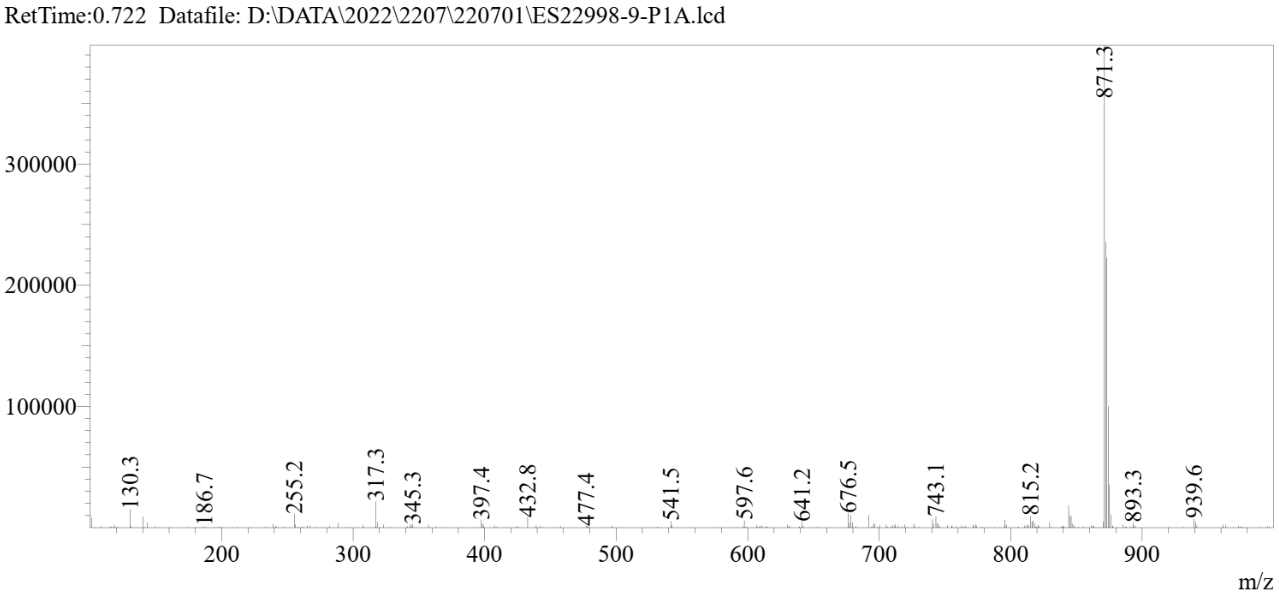

Supplementary Figure 3. ESI-MS spectrum of compound 4.

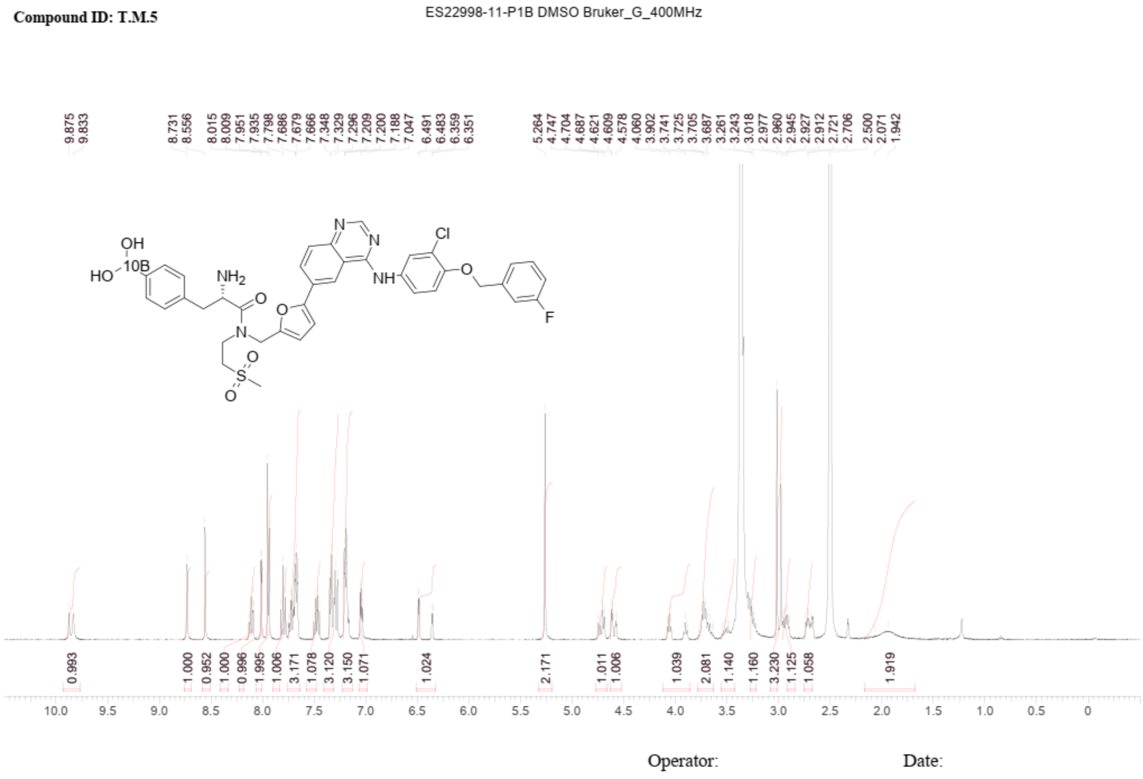

Supplementary Figure 4. ^{1}H-NMR spectrum of Lap-BPA in DMSO-d$_6$.

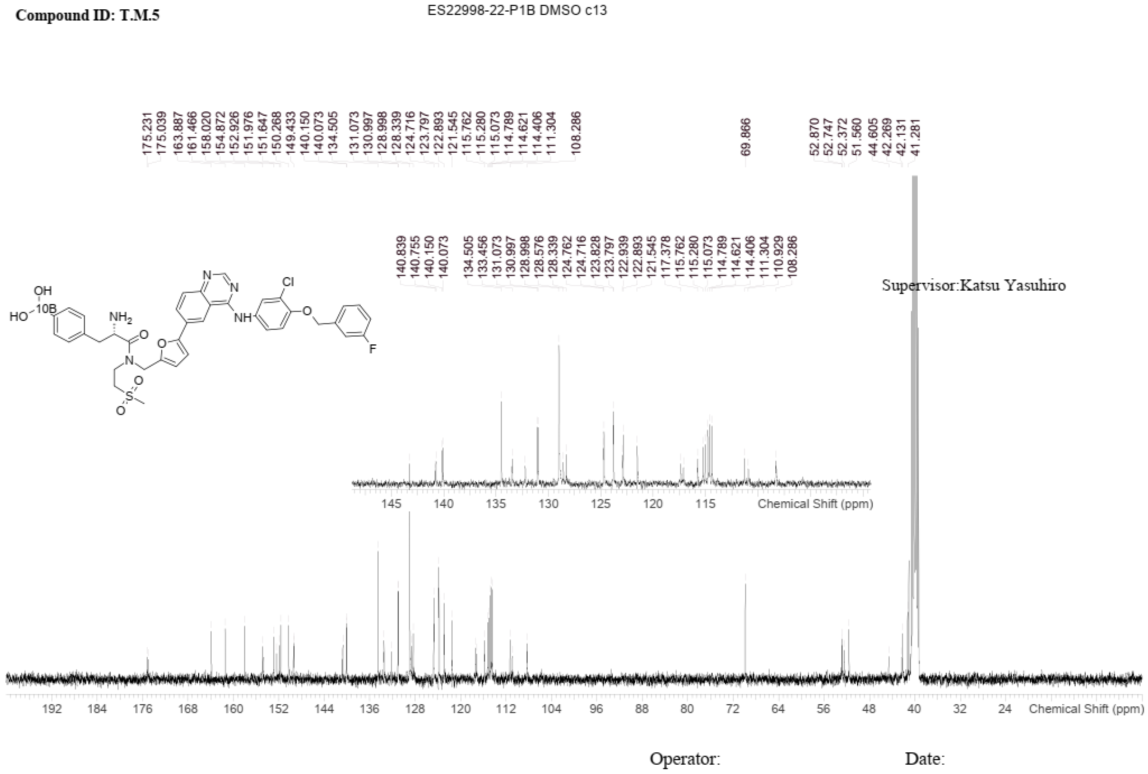

Supplementary Figure 5. ^{13}C-NMR spectrum of Lap-BPA in DMSO-d$_6$.

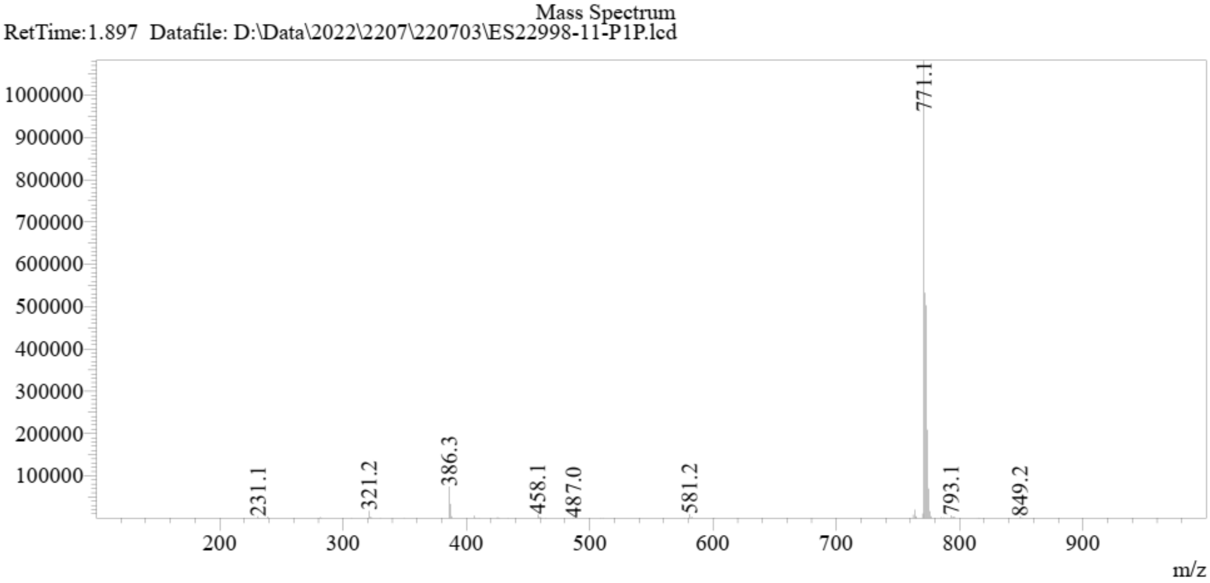

Supplementary Figure 6. ESI-MS spectrum of Lap-BPA.

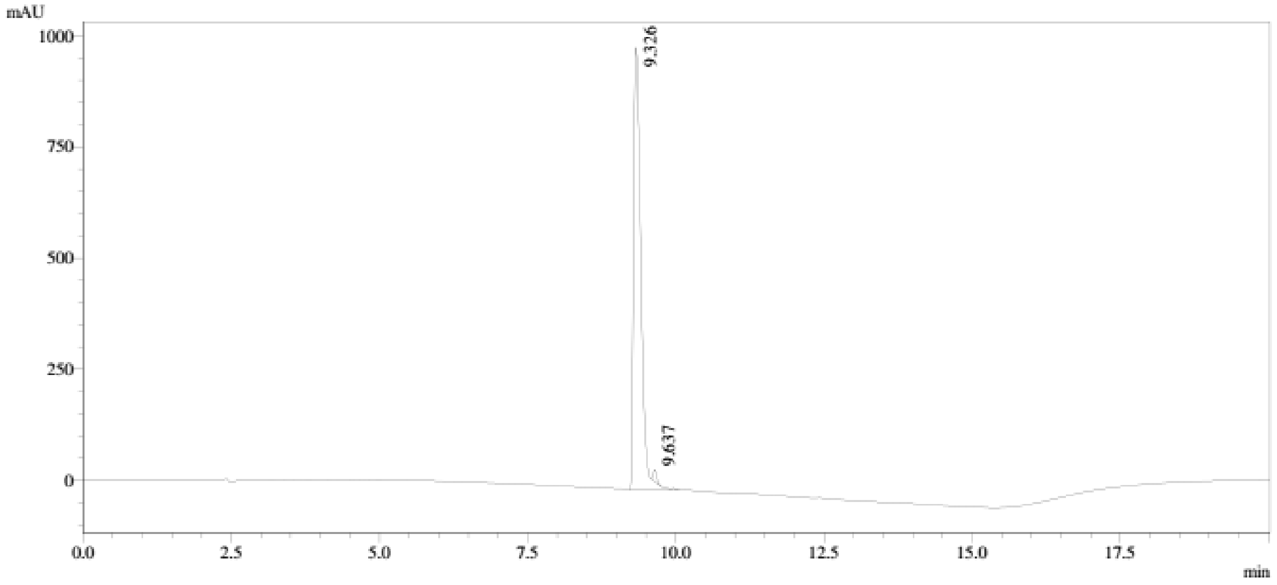

Integration Result

PDA Ch1 220nm 4nm

Peak#	Ret. Time	Area	Height	Area %
1	9.326	8541168	993816	98.820
2	9.637	102012	25791	1.180

Supplementary Figure 7. HPLC traces for Lap-BPA.

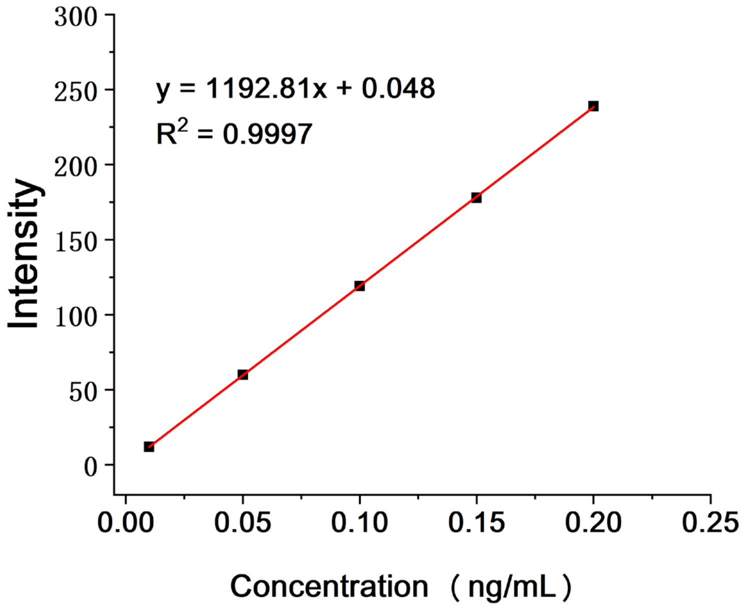

Supplementary Figure 8. Correlation curve between BPA concentration and absorption intensity.
